# Penetrating Foot Injury Caused by a Retained Wood Screw: A Case Report

**DOI:** 10.7759/cureus.102463

**Published:** 2026-01-28

**Authors:** Stanislaw Szymkiewicz

**Affiliations:** 1 Department of Urology, Janusz Korczak Provincial Specialist Hospital in Słupsk, Słupsk, POL

**Keywords:** case report, emergency department, penetrating foot injury, retained foreign body, wood screw

## Abstract

Penetrating foot injuries are associated with a significant risk of retained foreign bodies, infection, and damage to osseous or soft tissue structures. Imaging is routinely recommended to assess the depth of penetration and exclude bone involvement. We report a case of a penetrating foot injury caused by a metallic wood screw that penetrated the sole of the shoe and the plantar aspect of the foot. Clinical assessment by an orthopedic specialist and radiographic imaging revealed no evidence of osseous injury. The foreign body was removed under local anesthesia with intravenous analgesia using a controlled rotational technique, followed by empirical antibiotic therapy and tetanus prophylaxis. Post-procedural imaging confirmed complete removal of the foreign body. No immediate complications were observed in the emergency department, and the patient was discharged with an urgent referral for outpatient orthopedic follow-up. This case highlights the importance of combining careful clinical examination with appropriate imaging in the emergency department management of penetrating foot injuries to guide safe foreign body removal and reduce the risk of complications.

## Introduction

Penetrating foot injuries are common presentations in emergency departments and are associated with a significant risk of retained foreign bodies, secondary infection, and injury to soft tissue or osseous structures, particularly when the mechanism involves sharp or contaminated objects [1-4]. Such injuries may occur in occupational settings, construction sites, or household environments, where nails and wood screws are frequent causes of puncture wounds of the plantar surface.

The complex anatomy of the foot, including closely related bones, tendons, neurovascular structures, and deep plantar compartments, increases the risk that even small penetrating objects may cause clinically significant injury. In addition, penetration through footwear is associated with an increased risk of bacterial contamination and subsequent infection, including infections caused by Gram-negative organisms such as *Pseudomonas aeruginosa* [1,3,4].

Radiographic imaging is routinely recommended in the evaluation of penetrating foot injuries to assess the depth and trajectory of penetration, exclude osseous involvement, and identify retained foreign bodies, particularly when the penetrating object is metallic and radiopaque [1,2,4]. However, clinical examination remains essential for assessing neurovascular status, wound contamination, and the accessibility of the foreign body, all of which influence decisions regarding bedside removal versus operative management [1,3].

We report a case of a penetrating foot injury caused by a metallic wood screw that penetrated the sole of the shoe and the plantar aspect of the foot. The case highlights the role of combined clinical and radiographic assessment in guiding safe foreign body removal in the emergency department and illustrates practical considerations in the management of plantar puncture wounds.

## Case presentation

**Figure 1:**
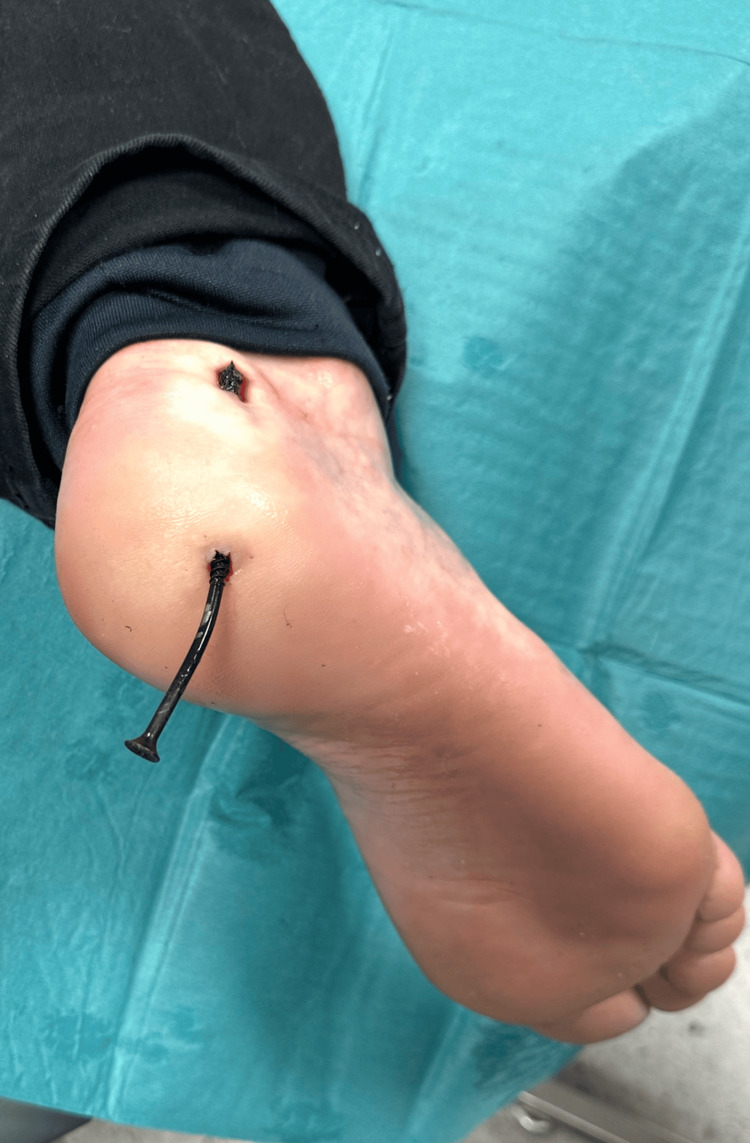
Clinical photograph showing a metallic wood screw penetrating the plantar aspect of the foot. The screw perforated the sole of the shoe and entered the plantar soft tissues. No active bleeding or gross contamination was visible at the time of examination.

The patient, with no known relevant comorbidities, presented to the emergency department shortly after injury and was triaged as a stable trauma patient requiring urgent evaluation. The injury occurred at a construction site when the patient jumped down from a stool approximately 50 cm in height and landed on a protruding metallic wood screw embedded in the floor, which penetrated the sole of the shoe and subsequently the plantar aspect of the foot (Figure 1). The patient reported localized pain without numbness or loss of function.

**Figure 2:**
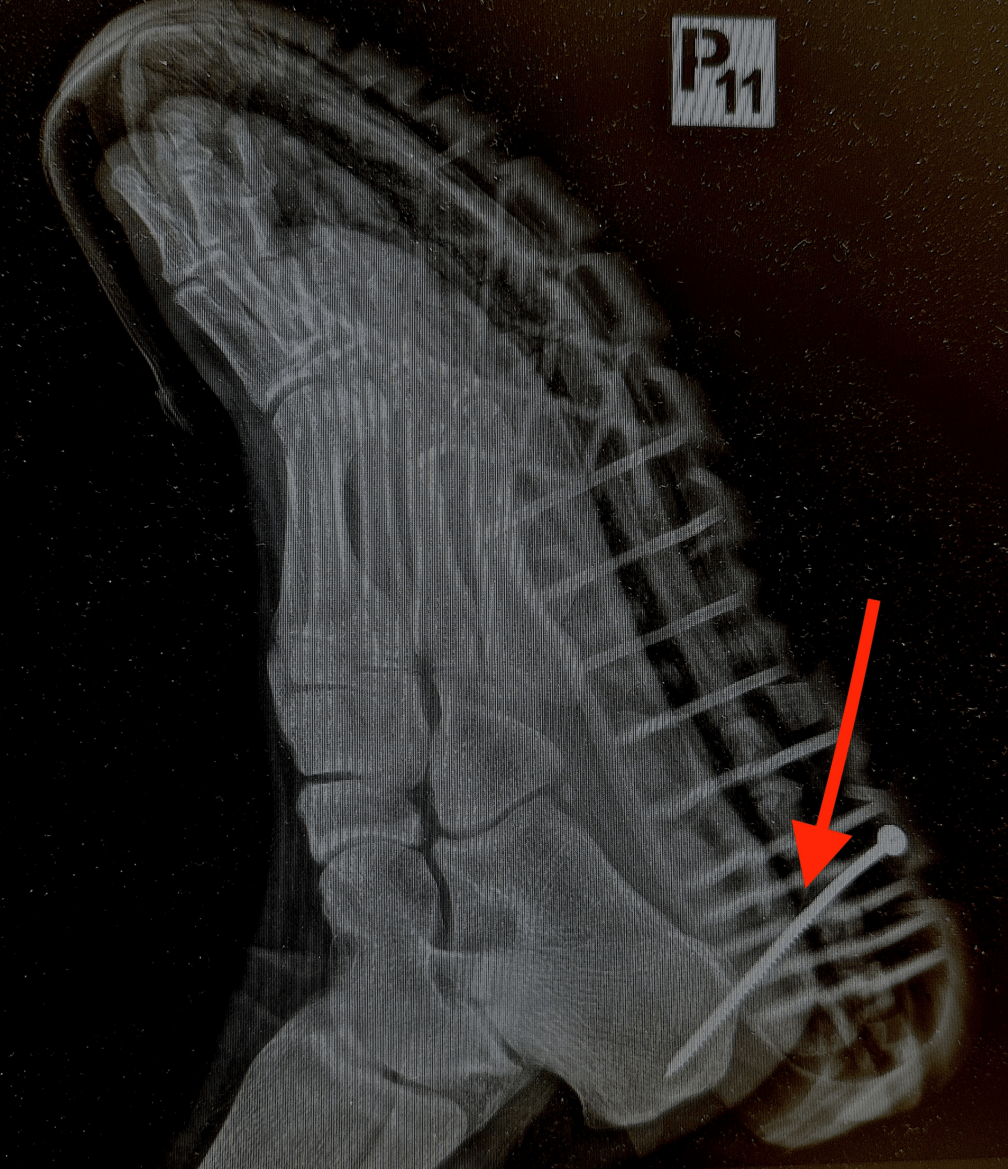
Pre-removal radiograph demonstrating wood screw penetration. Lateral radiograph of the foot showing a metallic wood screw penetrating the plantar soft tissues without evidence of osseous involvement. The red arrow indicates the position of the screw.

**Figure 3:**
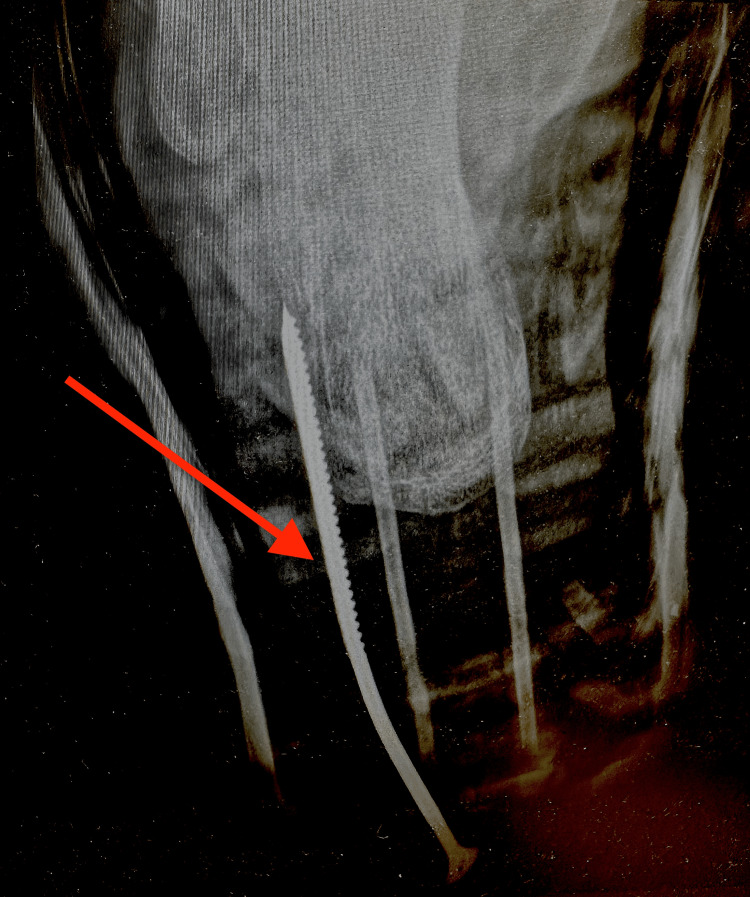
Additional pre-removal radiographic view of the wood screw trajectory. This radiographic projection demonstrates the metallic wood screw within the plantar soft tissues adjacent to the calcaneus, without evidence of bone injury. The red arrow indicates the position of the screw.

On physical examination, the screw was visibly penetrating the plantar aspect of the foot. There was localized tenderness without excessive bleeding, and no gross contamination was observed. Neurovascular status was intact. An orthopedic consultation was obtained, and plain radiography of the foot was performed to assess the depth and trajectory of penetration and to exclude osseous injury or retained fragments, in accordance with commonly recommended diagnostic approaches for penetrating foot injuries (Figures 2-3) [1,2,4].

**Figure 4:**
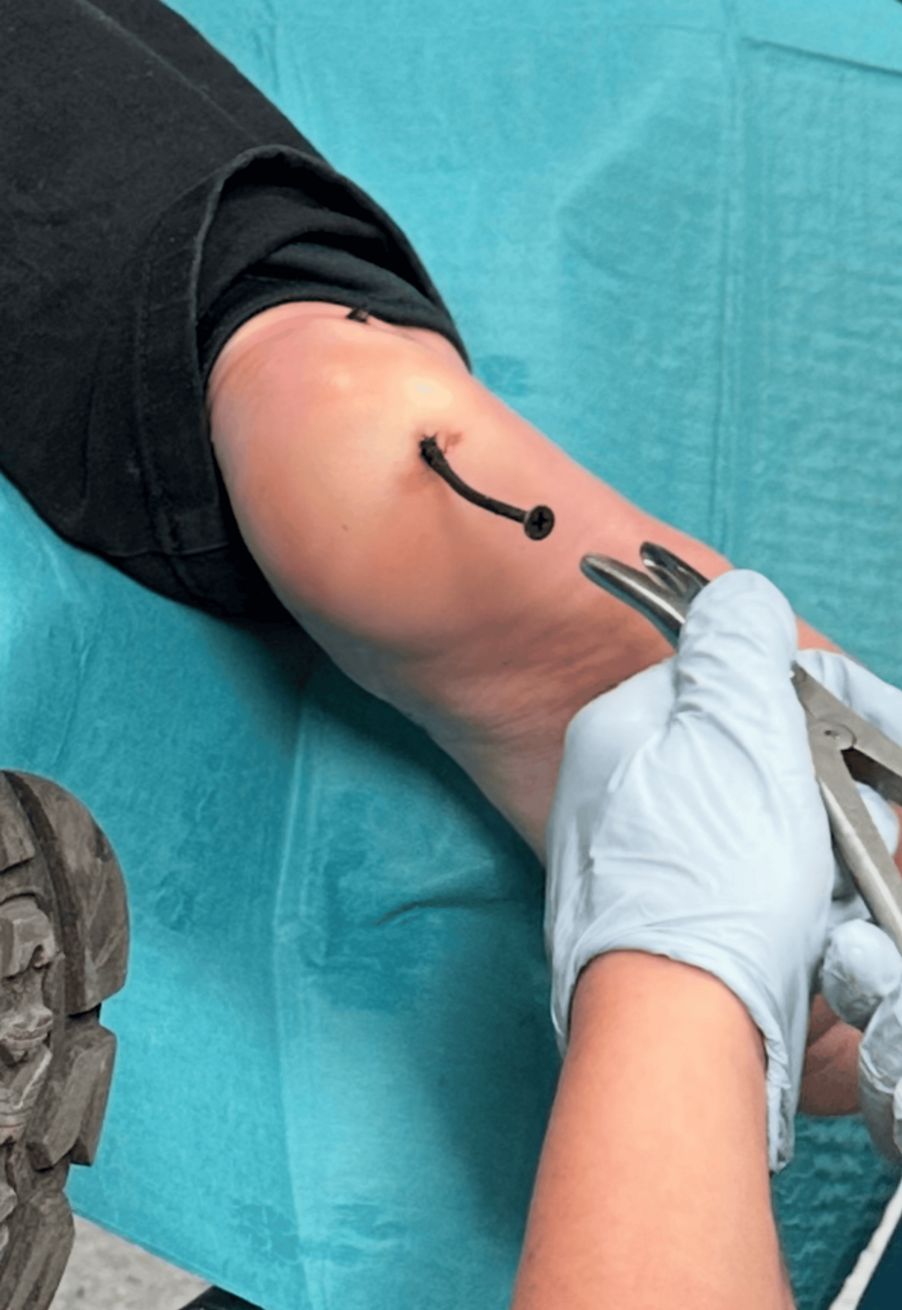
Intra-procedural photograph demonstrating controlled removal of a metallic wood screw from the plantar aspect of the foot using locking surgical forceps. The screw was extracted using a counterclockwise rotational technique under local anesthesia and intravenous analgesia.

The decision was made to proceed with the removal of the foreign body under controlled conditions in the emergency department. The patient received intravenous analgesia with fentanyl (100 µg) and metamizole (1 g), followed by local anesthesia using a 1% lidocaine solution. The screw was removed without resistance under direct visualization (Figure 4). No gross contamination or deep structural injury was observed during the procedure.

**Figure 5:**
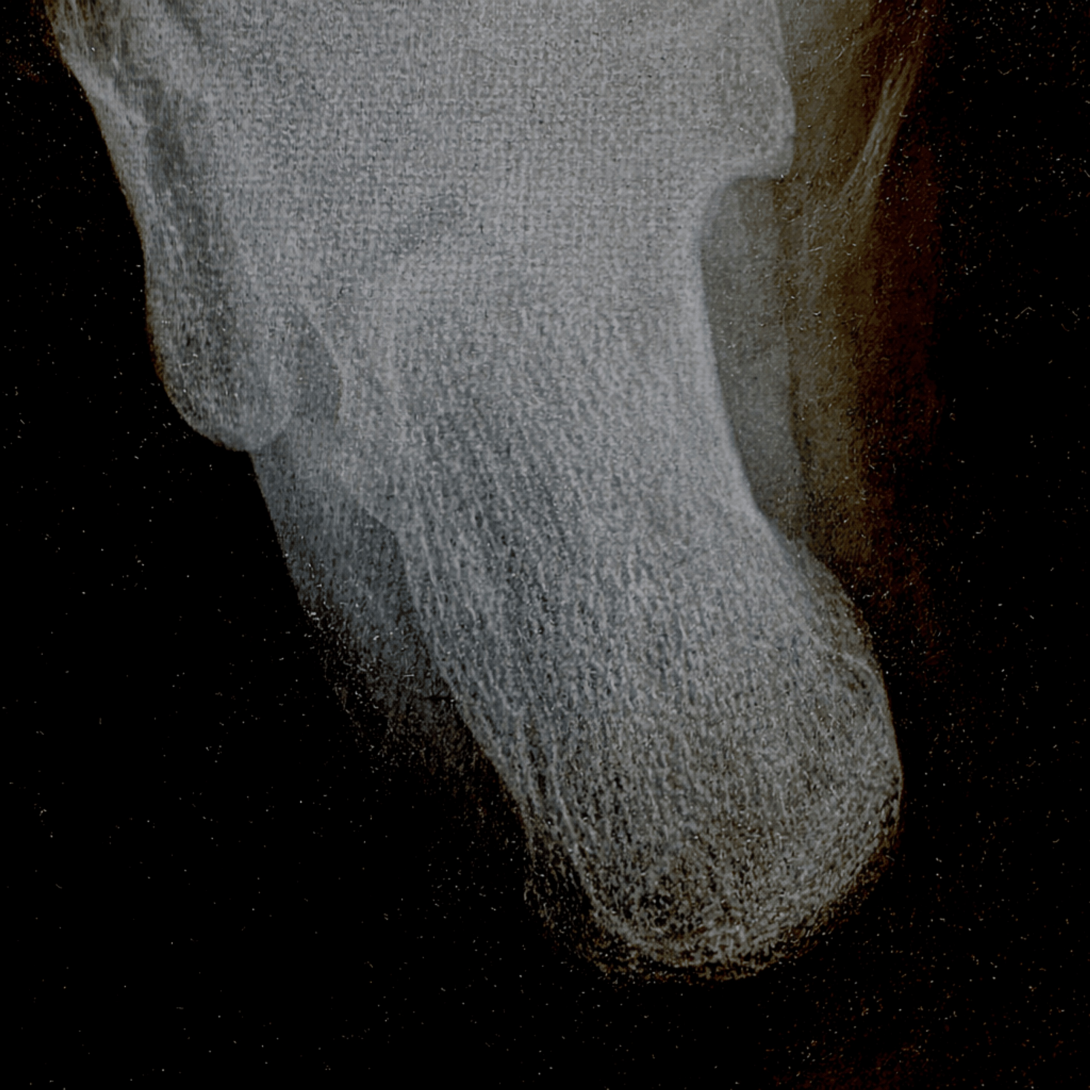
Post-removal radiograph of the foot. Post-procedural lateral radiograph demonstrating complete removal of the metallic wood screw, with no evidence of retained foreign body fragments or osseous injury.

**Figure 6:**
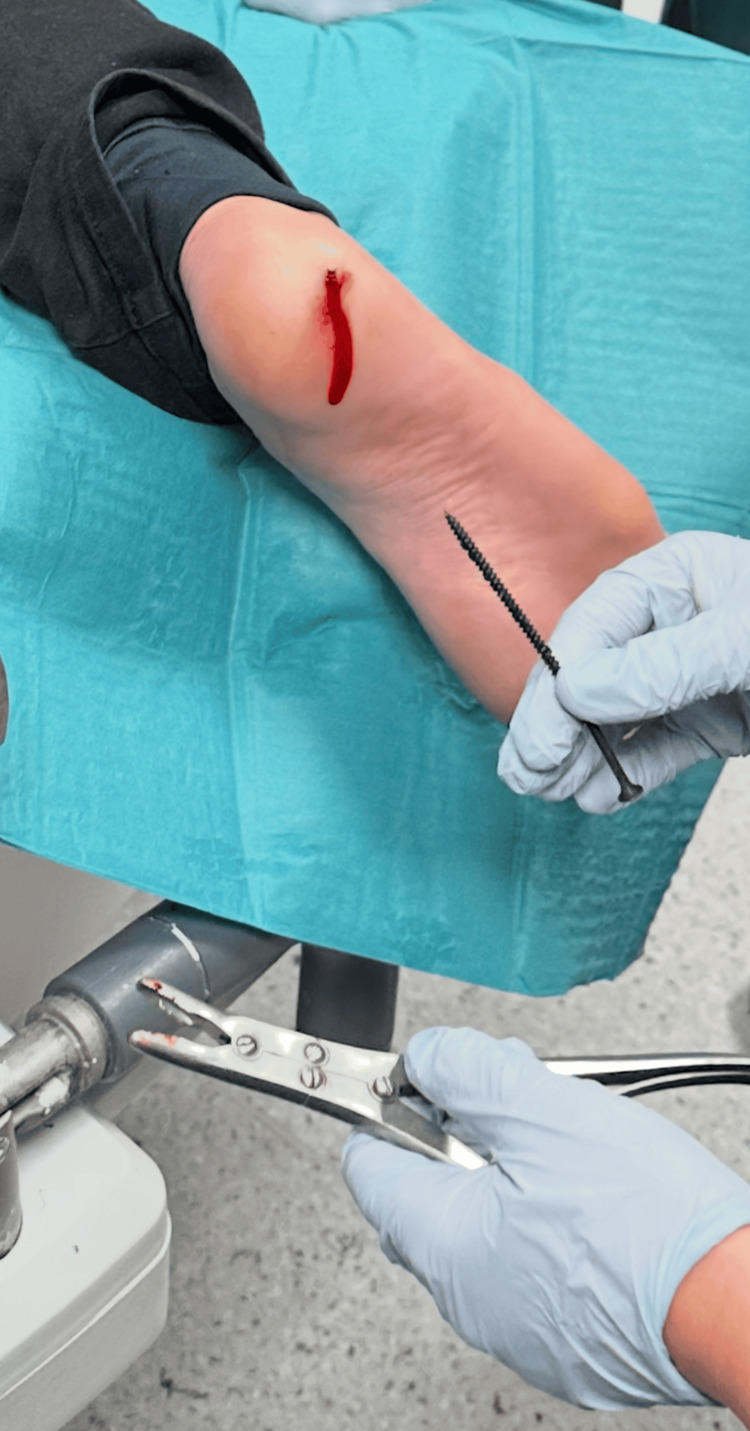
Post-procedural photograph showing the plantar wound immediately after removal of the metallic wood screw, with minimal bleeding at the entry site. The extracted screw is shown following successful rotational removal under local anesthesia and intravenous analgesia.

**Figure 7:**
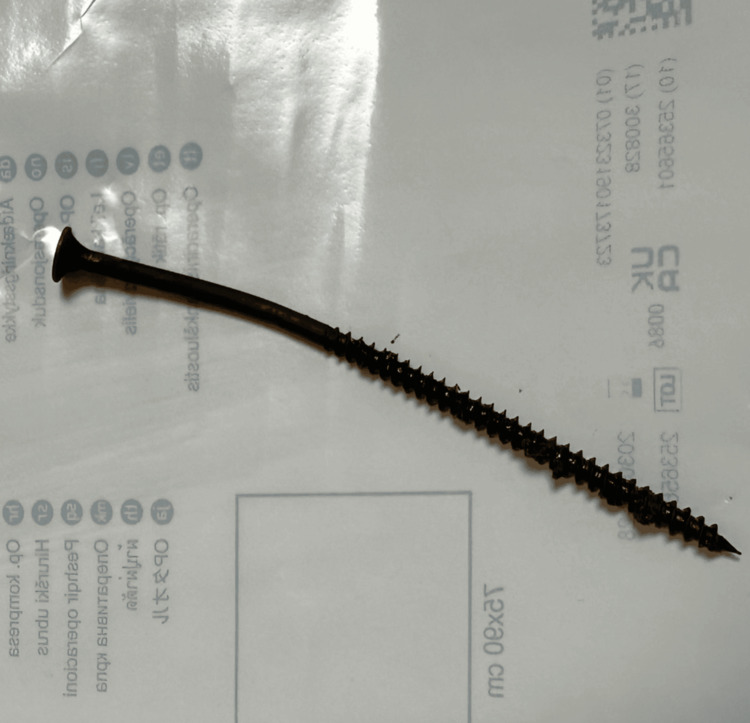
Photograph of the extracted metallic wood screw following removal from the plantar aspect of the foot. The length and threaded design of the screw illustrate the potential depth of penetration and the associated risk of soft tissue injury with this type of foreign body.

After removal, the wound was copiously irrigated with sterile normal saline, and a sterile dressing was applied (Figures 5-7). Given the penetrating mechanism through footwear and the associated risk of infection, empirical intravenous antibiotic therapy with ceftriaxone and metronidazole was administered. Tetanus prophylaxis was provided in the form of a tetanus toxoid booster (TT) only, as the patient had a documented complete vaccination history and did not meet criteria for tetanus immune globulin administration, consistent with standard recommendations for contaminated puncture wounds of the foot [1,3,4]. The patient tolerated the procedure well and was discharged home in stable condition with written instructions for wound care and an urgent referral for outpatient orthopedic follow-up. No immediate complications were observed, and no long-term follow-up data were available, as the patient did not return to the emergency department.

## Discussion

Penetrating foot injuries pose a diagnostic and therapeutic challenge due to the complex anatomy of the foot, the risk of infection, and the potential for occult osseous or tendon involvement. Radiographic imaging is routinely recommended in penetrating foot injuries to evaluate the depth and direction of penetration, exclude osseous involvement, and detect retained foreign bodies, particularly when the object is metallic and radiopaque [1,2,4]. In the present case, plain radiography in two projections demonstrated that the wood screw was confined to the plantar soft tissues and adjacent to the calcaneus without cortical disruption, which supported safe removal in the emergency department setting.

Nevertheless, careful clinical examination remains a critical component of initial assessment, particularly for evaluating neurovascular status, wound contamination, and the accessibility of the foreign body for potential bedside removal [1,3]. In this case, the combination of reassuring clinical findings and radiographic confirmation of the absence of bone involvement allowed for controlled bedside removal using a rotational technique under local anesthesia and intravenous analgesia.

Penetrating injuries of the foot that occur through footwear are associated with an increased risk of infection, including infections caused by Gram-negative organisms such as *P. aeruginosa*, which supports the use of empirical antibiotic therapy in selected cases [1,3,4]. Therefore, empirical antibiotic therapy and tetanus prophylaxis are commonly recommended, especially when contamination is suspected or follow-up may be uncertain. These measures were implemented in the present case to reduce the risk of delayed infectious complications.

Although exceedingly rare in the context of acute penetrating injuries, adverse tissue reactions related to metallic debris have been described in orthopedic literature, particularly in association with long-term metal implants and prosthetic components, a phenomenon known as metallosis [5]. In contrast, in the present case, the foreign body was promptly and completely removed, and post-procedural radiography confirmed the absence of retained metallic fragments, making such reactions highly unlikely. Nevertheless, this concept is mentioned to provide a broader context regarding potential tissue responses to metallic material when fragments remain in situ.

Selected penetrating foot injuries with clearly visualized foreign bodies, reassuring clinical findings, and no radiographic evidence of osseous involvement may be safely managed in the emergency department without operative exploration, provided that appropriate analgesia, sterile technique, and post-procedural care are ensured [1,2,4]. Prompt assessment, imaging, and multidisciplinary decision-making can facilitate timely and effective treatment while minimizing unnecessary delays.

In some cases, retained plantar foreign bodies may present with delayed or atypical symptoms, mimicking other foot pathologies such as plantar fasciitis, which may delay diagnosis and treatment [6]. This underscores the importance of maintaining a high index of suspicion and considering prior penetrating injury in patients presenting with persistent or unexplained plantar pain.

Previous case reports and series have described retained plantar foreign bodies presenting either acutely or with delayed symptoms, sometimes mimicking other foot pathologies and requiring operative exploration. However, most published cases focus on delayed diagnosis, infection, or surgical management rather than on immediate bedside treatment. In contrast, the present case demonstrates that even a long, threaded metallic foreign body located in close proximity to bone can be safely removed in the emergency department, provided that careful neurovascular assessment and multiplanar radiographic imaging confirm the absence of osseous involvement. The report also emphasizes the practical value of a controlled rotational extraction technique for threaded objects, which may minimize additional soft tissue trauma and facilitate successful bedside management.

## Conclusions

This case adds practical value by demonstrating that even long, threaded metallic foreign bodies penetrating the plantar aspect of the foot and lying in close proximity to bone can be safely removed in the emergency department, provided that careful neurovascular examination and multiplanar radiographic imaging confirm the absence of osseous involvement. The report also highlights the usefulness of a controlled rotational extraction technique for threaded objects, which may reduce additional soft tissue trauma compared with forceful traction. This case therefore supports a selective, imaging-guided bedside management approach for penetrating plantar injuries, potentially avoiding unnecessary operative exploration in appropriately selected patients.
